# Pure Red Blood Cell Aplasia and Isoniazid Use

**DOI:** 10.3201/eid1309.061334

**Published:** 2007-09

**Authors:** Pierre Loulergue, Olivier Mir, Robin Dhote

**Affiliations:** * Assistance Publique–Hôpitaux de Paris, Hôpital Necker, Paris, France; † Assistance Publique–Hôpitaux de Paris, Hôpital Cochin, Paris, France; ‡ Assistance Publique–Hôpitaux de Paris, Hôpital Avicenne, Bobigny, France

**Keywords:** isoniazid, pure red cell aplasia, anemia, tuberculosis, letter

**To the Editor:** Isoniazid is a first-line drug in the treatment of tuberculosis; its uses include prevention as well as cure. Isoniazid is usually well tolerated, although its common side effects include gastrointestinal discomfort, rash, allergy, hepatitis, and peripheral neuropathy. Hematologic disorders such as eosinophilia, thrombocytopenia, and autoimmune hemolytic anemia are rarely reported (*1*). Pure red cell aplasia (PRCA) is an uncommon disorder in adults. PRCA occurs secondary to drug exposure in 5% of patients; ≈30 drugs have been implicated (*2*), and few reports involving isoniazid have been published. We report a case of isoniazid-induced PRCA.

In 2006, a 79-year-old woman sought care at a public assistance hospital in Paris; she reported having had asthenia, breathlessness, and decreased appetite for 2 weeks. She had had node-negative, localized gastric adenocarcinoma 2 years earlier, which had been treated by partial gastrectomy, and pulmonary tuberculosis in 1948, which had been treated by partial pneumonectomy. As a result of a pleuropulmonary tuberculosis relapse diagnosed 6 weeks earlier, she was receiving antituberculous treatment with rifampin (400 mg/day), isoniazid (150 mg/day), ethambutol (800 mg/day), and pyrazinamide (1,000 mg/day). She received no other concomitant medications.

Her initial physical examination showed nothing abnormal. Laboratory analysis showed hemoglobin 6.3 g/dL, leukocyte and thrombocyte counts within normal limits, 1% reticulocytes, and zero schizocytes. Other test results (liver and renal function, serum folate and vitamin B12 levels, lactic dehydrogenase levels, C-reactive protein, serum protein electrophoresis, direct and indirect Coombs tests, and antinuclear antibody tests) were within normal limits, as were viral serologic test results (HIV, hepatitis B virus, hepatitis C virus, parvovirus B19). Upper and lower digestive tract endoscopic examination and carcinoembryonic antigen showed no abnormalities, which lessened the likelihood of a tumor relapse. Bone marrow aspiration from the sternum showed a hypocellular marrow with complete absence of the erythroid series, a normal myeloid series, and megakaryocytes. The marrow findings were consistent with PRCA.

In view of previous reports of isoniazid-induced PRCA (*3*–*5*), we suspected this drug to be responsible in this case. Isoniazid was withdrawn, and other antituberculous drugs were continued. The patient’s hemoglobin level rose to 10.6 g/dL and reticulocyte count to 3% over 10 days; no blood transfusion was required.

Drug-induced PRCA is a rare blood disorder in adults and has already been reported in isoniazid-treated patients (*3*–*5*). For this patient, other causes for anemia (e.g., drug-induced hemolytic anemia, digestive malignancies, viral causes known to date, hematologic malignancies, and autoimmune disorders) were excluded (*2*).

The favorable outcome after isoniazid withdrawal increased the likelihood that isoniazid was the cause. Although rechallenge with isoniazid could have confirmed isoniazid as the cause, it is not an ethical option because of the hazardous adverse effects.

The exact mechanism for isoniazid-induced PRCA remains unclear, but the demonstration of antibodies reacting with nucleated red blood cells in ≈50% of cases suggests an induction of autoimmunity (*4*,*5*). This hypothesis is supported by previously reported cases in which PRCA relapses occurred when treatment with isoniazid was resumed (*3*,*5*). The intrinsic imputability of isoniazid also relies on the lack of a dose-effect relationship and the delay between the introduction of isoniazid and the onset of PRCA, which can occur up to 6 months after start of treatment (*3*).

Clinicians treating patients with tuberculosis must be aware of this adverse reaction because failure to identify and discontinue isoniazid in patients with such a condition might lead to their illness and death. Given the ongoing worldwide HIV pandemic and the increase in tuberculosis it induces, such adverse effects are more likely to be reported in the next few years.
